# Family history of multiple sclerosis and long-term outcomes

**DOI:** 10.1007/s00415-026-14162-9

**Published:** 2026-09-26

**Authors:** Anna Karin Hedström, Tomas Olsson, Lars Alfredsson

**Affiliations:** 1 Department of Clinical Neuroscience, Karolinska Institutet, 17176 Stockholm, Sweden; 2 Institute of Environmental Medicine, Karolinska Institutet, Stockholm, Sweden; 3 Centre for Occupational and Environmental Medicine, Region Stockholm, Stockholm, Sweden

**Keywords:** Multiple sclerosis, Heredity, Disability progression

## Abstract

**Background:**

Family history is a well-established risk factor for multiple sclerosis (MS), but its influence on disease progression remains unclear. We investigated whether familial MS, including degree of relatedness, is associated with baseline characteristics and disease outcomes.

**Methods:**

We included individuals with incident MS from the Swedish population-based epidemiologic investigation of multiple sclerosis study with longitudinal follow-up through the Swedish MS registry. Participants were categorized as having no family history of MS, first-degree familial MS, or second-/third-degree familial MS. Outcomes included confirmed disability worsening (CDW), progression to Expanded Disability Status Scale milestones 3 and 4, cognitive worsening measured by the symbol digit modalities test, MRI disease activity, and worsening on the Multiple Sclerosis Impact Scale-29. Hazard ratios (HRs) with 95% confidence intervals (CIs) were estimated using Cox regression.

**Results:**

Among 3,094 participants, 239 (7.7%) reported a first-degree relative with MS and 468 (15.1%) reported a second-/third-degree relative with MS. Baseline characteristics were similar across groups, although HLA–DRB1*15:01 positivity was more common among individuals with first-degree familial MS. No significant associations were observed between familial MS status and disability progression, cognitive decline, MRI disease activity, or patient-reported worsening. Compared with individuals without a known family history of MS, the HR for CDW was 1.06 (95% CI 0.89–1.30) among individuals with first-degree familial MS and 1.04 (95% CI 0.90–1.20) among those with second-/third-degree familial MS.

**Conclusions:**

Family history of MS did not appear to have a substantial prognostic impact on long-term disease outcomes after MS onset.

**Supplementary Information:**

The online version contains supplementary material available at https://doi.org/10.1007/s00415-026-14162-9.

## Introduction

Multiple sclerosis (MS) is a chronic inflammatory and neurodegenerative disease of the central nervous system with a highly heterogeneous clinical course. While some individuals experience a relatively mild disease trajectory, others accumulate disability rapidly [1]. Identifying factors associated with long-term disease progression remains a major challenge and is essential for improving prognostication and guiding clinical management.

Genetic susceptibility plays a well-established role in the risk of developing MS [2]. Family studies have consistently demonstrated an increased risk among first-degree relatives [3, 4], and genome-wide association studies have identified more than 200 susceptibility loci, primarily related to immune function [2]. However, whether a family history of MS, also influences long-term disease course is less clear.

Previous studies of familial MS have yielded inconsistent findings regarding clinical characteristics and disease progression [5–10]. Some studies have suggested a more severe disease course among individuals with familial MS, including faster disability accumulation or a higher likelihood of progressive disease [5–9], whereas others have found few or no differences compared with sporadic MS [10]. Differences in age at onset, MRI findings, and genetic characteristics have also been described [11–13]. However, many studies have been limited by modest sample sizes, lack of detailed baseline data, or insufficient stratification by degree of relatedness.

In this study, we used data from a large, nationwide, population-based cohort with detailed baseline information and long-term follow-up to investigate whether family history of MS and degree of relatedness were associated with baseline demographic, clinical, environmental, or treatment-related characteristics. Among participants with relapsing-onset MS, we further examined long-term disease outcomes, including disability progression, cognitive decline, MRI activity, and patient-reported disease impact according to family history status.

## Methods

### Study design and study participants

This study included individuals with MS from the Swedish population-based case–control study Epidemiologic Investigation of Multiple Sclerosis (EIMS) [14]. Incident cases were recruited from 40 neurology units across Sweden, including all university hospitals, between April 2005 and December 2021 (*n* = 3792). Eligible participants were aged 16–70 years, resided in Sweden, and had a neurologist-confirmed diagnosis of MS according to the McDonald criteria. The participation rate was 91%. All participants completed a structured questionnaire at inclusion covering demographic characteristics, environmental exposures, lifestyle factors, and family history of MS.

Participants who reported that they did not know whether MS had occurred among their relatives were classified as having uncertain family history and were excluded from the primary analyses (*n* = 521), as were individuals without follow-up data on Expanded Disability Status Scale (EDSS) scores available through the Swedish MS registry (*n* = 177). The final analytic sample comprised 3094 individuals.

The study was approved by the Regional Ethical Review Board at Karolinska Institutet (reference number: 2004-252/1-4) and conducted in accordance with the Declaration of Helsinki and its later amendments. Participants provided written informed consent.

### Data collection

Family history of MS was assessed using self-reported questionnaire data at inclusion. Family history of MS was defined as MS in first-, second- or third-degree relatives. Participants were categorized into three groups according to degree of relatedness: no family history of MS, first-degree relatives with MS, and second- or third-degree relatives with MS. Participants were categorized hierarchically according to the closest affected relative. Those reporting at least one affected first-degree relative were classified in the first-degree group, irrespective of additional affected more distant relatives.

HLA–DRB1 and HLA-A alleles were determined at two-field resolution using the MS Replication Chip [2], an Illumina exome array supplemented with approximately 90,000 custom markers. HLA alleles were subsequently imputed using HLA*IMP:02 [15]. Participants were classified as carriers of or non-carriers of the respective alleles.

EBNA-1 antibody levels were assessed using multiplex serology to detect IgG antibodies against the EBNA-1 peptide segment (amino acids 385–420) [16], previously identified as the main MS-associated fragment [17]. Antibody levels were quantified using dual-laser flow-based detection and expressed as median fluorescence intensity (MFI).

Ancestry was classified as Nordic or non-Nordic. Individuals born in a Nordic country whose parents had not immigrated from outside the Nordic countries were classified as Nordic. Education was dichotomized as university education vs no university education. Smoking was dichotomized as current smoking vs non-smoking. A history of infectious mononucleosis (IM) was categorized as yes, no, or unsure. BMI at diagnosis was calculated using self-reported height and weight. Obesity was defined as BMI ≥30 kg/m^2^. A sun exposure index was constructed as a study-specific composite based on three questionnaire items reflecting habitual sun exposure, each scored 1–4, yielding an index ranging from 3 (lowest) to 12 (highest). Physical activity was assessed using four leisure-time activity levels and dichotomized into regular activity (exercise ≥1–2 times/week with sweating) and non-regular activity (predominantly sedentary activities or moderate activity without sweating). For patients recruited between 2005 and 2009, serum 25-hydroxy-vitamin D (ng/mL) was measured with a chemiluminescent immunoassay (Diasorin AB, Sweden) that detected both 25-hydroxy-vitamin D_2_ and D_3_.

### Outcomes

Clinical data were obtained from the Swedish MS Registry, which is used nationwide and integrated into routine clinical practice. Neurologists prospectively record information on disease activity, neurological function, and treatment exposure at each visit, enabling longitudinal assessment of disability outcomes.

Baseline was defined as the date of the first available EDSS assessment. Disability progression was assessed using confirmed disability worsening (CDW), defined as an increase in EDSS of at least 1.0 point from baseline, confirmed at a subsequent visit at least 6 months later (or ≥ 1.5 points if baseline EDSS was 0, and ≥ 0.5 points if baseline EDSS was ≥ 5.5). The first CDW event was further classified as relapse-associated worsening (RAW) if a relapse had occurred since the preceding EDSS assessment and as progression independent of relapse activity (PIRA) if no relapse had occurred during this interval. Time to confirmed EDSS milestones 3 and 4 was also analyzed. These analyses were restricted to participants with a baseline EDSS score below 3. MRI assessments were performed as part of routine clinical care, although the timing was not standardized. MRI disease activity was defined as the first occurrence of one or more new or enlarging T2 lesions on a follow-up MRI compared with the preceding scan. Cognitive outcomes were assessed using the symbol digit modalities test (SDMT). Cognitive worsening was defined as a decrease of 8 points or more from baseline [18]. Patient-reported disease impact was assessed using the Multiple Sclerosis Impact Scale-29 (MSIS-29), including both the physical and psychological subscales. Worsening was defined as an increase of 7.5 points or more from baseline on the respective subscale [19].

### Statistical analysis

Baseline characteristics were compared according to familial MS status using analysis of variance (ANOVA) for continuous variables and chi-square tests for categorical variables. Continuous variables are presented as means with standard deviations (SD) or medians with interquartile ranges (IQR), as appropriate.

Time-to-event analyses comprising participants with relapsing-onset MS were performed using Cox proportional hazards regression to estimate hazard ratios (HRs) with 95% confidence intervals (CIs) for disability progression, cognitive worsening, MRI activity, and patient-reported worsening according to familial MS status. RAW and PIRA were analyzed separately using cause-specific Cox models, with the alternative CDW type censored at the time of occurrence. Follow-up continued from baseline until occurrence of the outcome of interest, death, dropout, last available assessment for the respective outcome, or March 30, 2026, whichever occurred first. The proportional hazards assumption was assessed for all Cox regression models, with no evidence of violation.

Analyses were adjusted for age, sex, ancestry, education, calendar year of diagnosis, disease duration, baseline EDSS, smoking status, body mass index, and initial disease-modifying therapy (DMT) (none, platform therapy, or high-efficacy DMT). Disease duration was defined as the interval in years from MS onset to baseline and was included as a continuous variable. Platform therapy included interferon-beta, glatiramer acetate, teriflunomide, and dimethyl fumarate. High-efficacy DMTs included natalizumab, fingolimod, ocrelizumab, alemtuzumab, cladribine, mitoxantrone, and rituximab. Analyses of cognitive worsening were further adjusted for baseline SDMT score, while analyses of patient-reported outcomes were additionally adjusted for baseline MSIS-29 scores.

Additional longitudinal analyses of SDMT and MSIS-29 scores were performed using linear mixed-effects models with participant-specific random intercepts and slopes. Time was measured in years from the first assessment of the respective outcome. Models included family history group, time, and their interaction, and were adjusted for the covariates described above. To examine possible SDMT practice effects, test occasion, modeled as log2(occasion+1), and its interaction with family history were added to the model. For this analysis, family history was dichotomized as any vs no family history.

Sensitivity analyses included models with disease-modifying therapy as a time-varying covariate and models in which participants with uncertain family history of MS were included in the non-familial MS group. In further sensitivity analyses, second- and third-degree family history were analyzed separately, with participants reporting affected relatives of different degrees classified according to their closest affected relative. Finally, an additional sensitivity analysis was performed using a decrease of at least 4 points in the SDMT score as the definition of cognitive worsening. All statistical analyses were performed using SAS software version 9.4 (SAS Institute, Cary, NC, USA). All tests were two-sided, and *p* values < 0.05 were considered statistically significant.

## Results

A total of 3,094 individuals with MS were included in the study, of whom 239 (7.7%) reported a first-degree relative with MS and 468 (15.1%) reported a second- or third-degree relative with MS. Individuals with second-/third-degree familial MS were slightly younger at diagnosis and study inclusion compared with those without a known family history of MS, whereas no significant differences were observed for age at onset, sex distribution, or MS phenotype. The frequency of HLA–DRB1*15:01 positivity was higher among individuals with first-degree familial MS compared with the other groups (68.3% vs. 52.8% and 53.4%, *p* = 0.005). Otherwise, baseline clinical, environmental, and treatment-related characteristics were broadly similar across groups (Table 1).

**Table 1:** Characteristics of study population

Characteristics		Total	Familial MS status			*p* value
			First-degree relative	Second-/third-degree relative	No known family history	
*N*		3094	239	468	2387	
Age at onset (SD)		34.3 (10.6)	34.3 (10.8)	33.4 (10.3)	34.4 (10.6)	0.13
Age at diagnosis (SD)		37.4 (11.1)	37.7 (11.2)	36.2 (10.9)	37.6 (11.2)	0.04
Mean time to diagnosis (SD)		3.1 (5.3)	3.4 (5.5)	2.8 (5.0)	3.1 (5.3)	0.28
Age at study inclusion (SD)		37.9 (11.2)	38.5 (11.4)	36.7 (10.8)	38.1 (11.2)	0.03
Disease duration at baseline (IQR)		1.5 (0.4–4.8)	1.7 (0.6–6.0)	1.4 (0.4–4.7)	1.5 (0.4–4.7)	0.13
Female, *n* (%)		2198 (71.0)	170 (71.1)	341 (72.9)	1687 (70.7)	0.63
Nordic, *n* (%)		2546 (82.3)	197 (82.4)	392 (83.8)	1957 (82.0)	0.65
MS type, *n* (%)	Relapsing onset	2879 (93.1)	222 (92.9)	449 (95.9)	2208 (92.5)	0.11
	Progressive onset	115 (3.7)	10 (4.2)	9 (1.9)	96 (4.0)	
	Unknown	100 (3.2)	7 (2.9)	10 (2.1)	83 (3.5)	
OCB, *n* (%)		1382 (91.3)	113 (91.1)	207 (93.2)	1062 (90.9)	0.53
Time to DMT initiation, years (SD)		1.5 (2.5)	1.5 (2.5)	1.4 (2.2)	1.6 (2.6)	0.13
Treatment, *n* (%)	None	122 (3.9)	8 (3.4)	16 (3.4)	98 (4.1)	0.67
	Platform therapy	560 (18.1)	47 (19.7)	87 (18.6)	426 (17.9)	
	Escalation	1542 (49.8)	123 (51.5)	239 (51.1)	1180 (49.4)	
	High-efficacy DMT	870 (28.1)	61 (25.5)	126 (26.9)	683 (28.6)	
Proportion of follow-up on DMT (SD)		0.8 (0.3)	0.8 (0.3)	0.8 (0.3)	0.8 (0.3)	0.43
MRI assessments per year (IQR)		0.9 (0.6–1.5)	0.9 (0.6–1.6)	0.9 (0.6–1.8)	0.9 (0.6–1.4)	0.17
Baseline EDSS, median (IQR)		1.5 (1.0–2.5)	1.5 (1.0–2.5)	1.5 (1.0–2.5)	1.5 (1.0–2.5)	0.68
Baseline SDMT, median (IQR)		53 (46–60)	53 (47–59)	54 (47–60)	53 (45–60)	0.29
HLA–DRB1*15:01, *n* (%)		881 (54.5)	86 (68.3)	123 (52.8)	672 (53.4)	0.005
HLA-A*02:01, *n* (%)		660 (40.8)	46 (36.5)	102 (43.8)	512 (40.7)	0.41
EBNA-1 antibody levels		7701 (2990)	7229 (3270)	7468 (3209)	7792 (2921)	0.31
University education, *n* (%)		1463 (47.3)	102 (42.7)	222 (47.4)	1139 (47.7)	0.43
IM, *n* (%)	Yes	492 (15.9)	32 (13.4)	67 (14.3)	393 (16.5)	0.40
	No	2241 (72.4)	170 (71.1)	350 (74.8)	1721 (72.1)	
	Unsure	361 (11.7)	37 (15.5)	51 (10.9)	273 (11.4)	
Current smoking, *n* (%)		631 (20.4)	52 (21.8)	100 (21.4)	479 (20.1)	0.70
Body mass index (SD)		25.1 (4.9)	24.7 (5.0)	24.7 (4.5)	25.2 (5.0)	0.51
Sun exposure index (SD)		6.2 (1.8)	6.2 (1.9)	6.2 (1.9)	6.1 (1.8)	0.50
Vitamin D levels		61.6 (27.0)	65.4 (24.9)	63.4 (25.5)	61.1 (27.5)	0.40
Regular physical activity, *n* (%)		1228 (39.7)	90 (37.7)	200 (42.7)	938 (39.3)	0.30

Escalation represents initiation with platform therapy and subsequent escalation to high-efficacy DMT

DMT, disease-modifying therapy; OCB, oligoclonal bands; EDSS, expanded disability status scale; SDMT, symbol digit modalities test; HLA, human leukocyte antigen; EBNA, Epstein–Barr nuclear antigen; IM, infectious mononucleosis; SD, standard deviation; IQR, interquartile range

During follow-up, no significant associations were observed between familial MS status and disability progression outcomes (Table 2). Compared with individuals without a known family history of MS, the HR for CDW was 1.06 (95% CI 0.89–1.30) among individuals with a first-degree relative with MS and 1.04 (95% CI 0.90–1.20) among those with a second-/third-degree relative with MS. Separate analyses of the components of CDW also showed no clear associations with familial MS status (Table 2). Results remained similar when DMT exposure was modeled as a time-varying covariate, with HRs for CDW of 1.09 (95% CI 0.97–1.23) among individuals with a first-degree relative with MS and 0.95 (95% CI 0.84–1.04) among those with a second- or third-degree relative with MS (eTable 1). Similarly, no clear associations were observed for progression to EDSS 3 or EDSS 4. Inclusion of participants with uncertain family history of MS in the non-familial MS group yielded similar results (eTable 2). When second- and third-degree family history were analyzed separately, no consistent associations or gradient according to degree of relatedness were observed across the outcomes (eTable 3).

**Table 2:** Disability outcomes according to familial MS status

Familial MS status	*N*	Years (SD)	Events (%)	HR (95% CI)^1^	HR (95% CI)^2^
*CDW*					
No known family history	2208	6.8 (5.4)	1046 (47.4)	1.0 (reference)	1.0 (reference)
First-degree relative	222	6.0 (4.7)	113 (50.9)	1.14 (0.95–1.38)	1.06 (0.89–1.30)
Second-/third-degree relative	449	7.2 (5.5)	223 (49.7)	1.02 (0.89–1.18)	1.04 (0.90–1.20)
*RAW*					
No known family history	2208	6.8 (5.4)	270 (12.2)	1.0 (reference)	1.0 (reference)
First-degree relative	222	6.0 (4.7)	28 (12.6)	1.11 (0.75–1.64)	1.05 (0.71–1.55)
Second-/third-degree relative	449	7.2 (5.5)	50 (11.1)	0.90 (0.67–1.20)	0.94 (0.70–1.25)
*PIRA*					
No known family history	2208	6.8 (5.4)	776 (35.1)	1.0 (reference)	1.0 (reference)
First-degree relative	222	6.0 (4.7)	85 (38.3)	1.14 (0.92–1.44)	1.10 (0.87–1.39)
Second-/third-degree relative	449	7.2 (5.5)	173 (38.5)	1.07 (0.91–1.26)	1.08 (0.92–1.28)
*EDSS 3*					
No known family history	1759	8.4 (5.7)	548 (31.2)	1.0 (reference)	1.0 (reference)
First-degree relative	187	7.3 (5.1)	64 (34.2)	1.17 (0.88–1.52)	1.16 (0.87–1.49)
Second-/third-degree relative	359	8.6 (5.6)	117 (32.6)	1.01 (0.81–1.27)	1.05 (0.86–1.29)
*EDSS 4*					
No known family history	1759	9.9 (5.8)	257 (14.6)	1.0 (reference)	1.0 (reference)
First-degree relative	187	9.2 (5.5)	25 (13.4)	1.01 (0.67–1.53)	0.97 (0.64–1.46)
Second-/third-degree relative	359	10.2 (5.5)	47 (13.1)	0.91 (0.67–1.24)	0.89 (0.65–1.22)

CDW, confirmed disability worsening; RAW, relapse-associated worsening; PIRA, progression independent of relapse activity; EDSS, expanded disability status scale; HR, hazard ratio; CI, confidence interval; BMI, body mass index; DMT, disease-modifying therapy

^1^Adjusted for age and sex

^2^Adjusted for age, sex, ancestry, education, calendar year of diagnosis, disease duration, baseline EDSS, smoking, BMI, and DMT exposure

No significant associations were observed between familial MS status and MRI disease activity (Table 3). Compared with individuals without a known family history of MS, the HR for new MRI lesions was 1.11 (95% CI 0.92–1.35) among individuals with first-degree familial MS and 0.95 (95% CI 0.82–1.09) among those with second-/third-degree familial MS.

**Table 3:** MRI disease activity according to familial MS status

Familial MS status	*N*	Years (SD)	Events (%)	HR (95% CI)^1^	HR (95% CI)^2^
*New MRI lesions*					
No known family history	2081	5.9 (5.4)	1156 (55.6)	1.0 (reference)	1.0 (reference)
First-degree relative	201	5.7 (5.4)	116 (57.7)	1.12 (0.92–1.36)	1.11 (0.92–1.35)
Second-/third-degree relative	422	5.9 (4.9)	227 (53.6)	0.94 (0.82–1.09)	0.95 (0.82–1.09)

MRI, magnetic resonance imaging; HR, hazard ratio; CI, confidence interval; BMI, body mass index; DMT, disease-modifying therapy

^1^Adjusted for age and sex

^2^Adjusted for age, sex, ancestry, education, calendar year of diagnosis, disease duration, baseline EDSS, smoking, BMI, and DMT exposure

Familial MS status was also not associated with cognitive worsening measured by the SDMT (Table 4). The HR for cognitive worsening was 1.05 (95% CI 0.79–1.41) among individuals with first-degree familial MS and 0.93 (95% CI 0.75–1.15) among those with second-/third-degree familial MS. In a sensitivity analysis defining cognitive worsening as a decrease of at least 4 SDMT points, the corresponding HRs were 1.14 (95% CI 0.92–1.42) for first-degree family history and 0.95 (95% CI 0.80–1.11) for second-/third-degree family history. Similarly, no significant associations were observed between familial MS status and patient-reported worsening on either the physical or psychological MSIS-29 subscales (Table 5). Additional mixed-effects analyses showed no evidence that longitudinal changes differed among the family history groups for SDMT (*P* for interaction with time = 0.328), MSIS-29 physical scores (*P* = 0.742), or MSIS-29 psychological scores (*P* = 0.941) (eTable 4). When family history was dichotomized as any vs none, there was no evidence that the association between test occasion and SDMT change differed by family history status (*P* = 0.267).

**Table 4:** Cognitive decline according to familial MS status

Familial MS status	*N*	Years (SD)	Events (%)	HR (95% CI)^1^	HR (95% CI)^2^
*SDMT*					
No known family history	1816	6.6 (4.1)	526 (29.0)	1.0 (reference)	1.0 (reference)
First-degree relative	183	5.9 (3.9)	50 (27.3)	1.05 (0.79–1.40)	1.05 (0.79–1.41)
Second-/third-degree relative	385	6.6 (4.0)	100 (26.0)	0.93 (0.75–1.15)	0.93 (0.75–1.15)

SDMT, symbol digit modalities test; HR, hazard ratio; CI, confidence interval; BMI, body mass index; DMT, disease-modifying therapy

^1^Adjusted for age and sex

^2^Adjusted for age, sex, ancestry, education, calendar year of diagnosis, disease duration, baseline EDSS, smoking, BMI, baseline SDMT score, and DMT exposure

**Table 5:** Patient-reported disease impact according to familial MS status

Familial MS status	*N*	Years (SD)	Events (%)	HR (95% CI)^1^	HR (95% CI)^2^
*MSIS-29 physical*					
No known family history	1894	6.3 (5.1)	862 (45.5)	1.0 (reference)	1.0 (reference)
First-degree relative	195	5.5 (5.1)	82 (42.3)	1.05 (0.84–1.31)	1.01 (0.80–1.26)
Second-/third-degree relative	397	6.2 (4.9)	169 (42.6)	0.97 (0.82–1.15)	1.01 (0.85–1.19)
*MSIS-29 psychological*					
No known family history	1894	5.7 (5.1)	1016 (53.7)	1.0 (reference)	1.0 (reference)
First-degree relative	195	5.1 (5.0)	101 (51.8)	1.06 (0.86–1.30)	1.06 (0.86–1.26)
Second-/third-degree relative	397	5.9 (4.8)	196 (49.6)	0.89 (0.77–1.04)	0.93 (0.80–1.09)

MSIS, multiple sclerosis impact scale; HR, hazard ratio; CI, confidence interval; BMI, body mass index; DMT, disease-modifying therapy

^1^Adjusted for age and sex

^2^Adjusted for age, sex, ancestry, education, calendar year of diagnosis, disease duration, baseline EDSS, smoking, BMI, baseline MSIS-29 score; and DMT exposure

## Discussion

In this large population-based study of individuals with incident MS, baseline demographic, clinical, environmental, and treatment-related characteristics were broadly similar across familial MS groups, although individuals with first-degree familial MS had a higher frequency of HLA–DRB1*15:01 positivity. Among cases with relapsing-onset MS, we found no evidence that family history of MS, including degree of relatedness, was associated with disability progression, cognitive decline, MRI disease activity, or patient-reported worsening. Our findings suggest that although familial predisposition is an established determinant of susceptibility to MS, it does not appear to substantially influence disease severity or progression after disease onset.

Our results add to a literature that has been inconsistent regarding the role of family history in MS prognosis. While genetic susceptibility is well-established in MS etiology, with increased risk among relatives and more than 200 identified susceptibility loci [2], whether familial predisposition also influences disease course has remained unclear. Some previous studies have suggested that familial MS may be associated with a more progressive phenotype or a less favorable prognosis [5–9], whereas others have reported little or no association with disability outcomes [10, 20]. Our findings support the latter and suggest that family history of MS, including degree of relatedness, has limited prognostic relevance once the disease has developed.

Although many genetic variants contribute to susceptibility to MS [2], the mechanisms underlying disease initiation may differ from those driving neurodegeneration, progression, and long-term disability accumulation after disease onset. Most established MS susceptibility loci are related to immune regulation and risk of developing disease rather than subsequent disease severity [21]. It is, therefore, possible that familial clustering primarily reflects shared predisposition to disease initiation, while later disease progression is more strongly influenced by other factors, including treatment exposure, aging, comorbidities, and lifestyle-related factors.

The absence of differences in baseline clinical characteristics further supports this interpretation. We observed no clear differences in age at onset, MS phenotype, baseline disability measures, or relevant environmental and lifestyle-related factors, nor in biological markers, such as EBNA-1 antibody levels and vitamin D levels. This overall similarity across the groups suggests that individuals with and without a family history of MS constitute clinically and epidemiologically similar populations at disease onset despite differences in inherited susceptibility.

Another possible explanation for the null findings is that family history, although clinically intuitive, is a relatively crude measure of inherited susceptibility. It does not capture the underlying burden or composition of genetic risk variants and may, therefore, obscure more specific genetic influences on disease progression. Family history also reflects shared familial environment in addition to inherited factors. Consequently, individuals classified as having familial MS may differ substantially in their underlying genetic risk profiles, while individuals without a known family history may still carry considerable genetic susceptibility. Such heterogeneity would tend to attenuate associations. Our findings, therefore, do not exclude the possibility that specific genetic variants or gene–environment interactions may influence disease progression [22], even if reported family history itself has limited prognostic value.

Several limitations should be considered. Family history was self-reported and may, therefore, be subject to incomplete knowledge of disease among relatives. This is particularly relevant for more distant relatives for whom participants may have less detailed knowledge, and could have introduced nondifferential misclassification, potentially biasing estimates toward the null. However, analyses separating first-, second-, and third-degree relatives did not reveal a consistent pattern suggestive of a true underlying association. Family history is also an indirect marker of inherited susceptibility and may not adequately capture the relevant biological variation underlying progression. Residual confounding is also possible. Depression could not be included as a covariate, because no separate validated measure was available. Nevertheless, the absence of differences in measured baseline characteristics argues against major confounding by the factors assessed here.

Follow-up duration differed somewhat across outcomes, and MRI, cognitive, and patient-reported measures were not available for all participants. Although these analyses remained broadly consistent with the primary disability outcomes, some degree of selection bias cannot be excluded. In addition, EDSS-based outcomes primarily capture ambulatory disability and may be less sensitive to other dimensions of disease progression. While we adjusted for treatment exposure and performed sensitivity analyses using time-varying DMT exposure, treatment decisions in observational studies are influenced by disease activity and clinical characteristics, and residual treatment-related confounding may, therefore, remain. Although MRI assessments were performed according to routine clinical practice and not at standardized intervals, their frequency was similar across family history groups, reducing the likelihood that differential surveillance influenced the comparisons. Finally, although the study was large overall, the number of individuals with first-degree familial MS was comparatively modest for some outcomes, limiting statistical power to detect small associations.

In conclusion, family history of MS did not appear to have a substantial prognostic impact on long-term disease outcomes after MS onset. However, modest associations, particularly among individuals with first-degree familial MS, cannot be excluded.

## Supplementary Information

Below is the link to the electronic supplementary material.

Supplementary file1 (DOCX 21 KB)

Supplementary file2 (DOCX 35 KB)

## Data Availability

Anonymized data underlying this article will be shared on reasonable request from any qualified investigator that wants to analyze questions that are related to the published article.
