# Supplementary material for: Family history of multiple sclerosis and long-term outcomes
Source: J Neurol. 2026 Sep 26;273(10):622. doi: 10.1007/s00415-026-14162-9 (PMC13615947; doi:10.1007/s00415-026-14162-9)
Supplement: Supplementary file 1 [file 415_2026_14162_MOESM1_ESM.docx]

eTable 1. Sensitivity analyses with DMT as a time-varying variable: disability outcomes according to familial MS status

| CDW | | | | | |
| --- | --- | --- | --- | --- | --- |
| Heredity | N | Years (SD) | Outcome (%) | HR (95% CI)^1^ | HR (95% CI)^2^ |
| No known family history | 2208 | 6.8 (5.4) | 1046 (47.4) | 1.0 (reference) | 1.0 (reference) |
| 1st degree | 222 | 6.0 (4.7) | 113 (50.9) | 1.10 (0.91-1.33) | 1.09 (0.97-1.23) |
| 2-3rd degree | 449 | 7.2 (5.5) | 223 (49.7) | 1.00 (0.87-1.16) | 0.95 (0.84-1.04) |

CDW=confirmed disability worsening; EDSS=expanded disability status scale; HR=hazard ratio; CI=confidence interval; BMI=body mass index; DMT=disease-modifying therapy; ^1^adjusted for age and sex; ^2^adjusted for age, sex, ancestry, education, calendar year of diagnosis, disease duration, baseline EDSS, smoking, BMI, and DMT exposure.

eTable 2. Sensitivity analyses including participants with uncertain family history: disability outcomes according to familial MS status

| CDW | | | | | |
| --- | --- | --- | --- | --- | --- |
| Heredity | N | Years (SD) | Outcome (%) | HR (95% CI)^1^ | HR (95% CI)^2^ |
| No known family history | 2692 | 6.9 (5.4) | 1298 (48.2) | 1.0 (reference) | 1.0 (reference) |
| 1st degree | 222 | 6.2 (4.8) | 113 (50.9) | 1.10 (0.91-1.33) | 1.05 (0.86-1.27) |
| 2-3rd degree | 449 | 7.3 (5.5) | 223 (49.7) | 1.00 (0.87-1.16) | 1.03 (0.89-1.19) |
| EDSS 3 | | | | | |
| Heredity | N | Years (SD) | Outcome (%) | HR (95% CI)^1^ | HR (95% CI)^2^ |
| No known family history | 2147 | 8.4 (5.8) | 696 (32.5) | 1.0 (reference) | 1.0 (reference) |
| 1st degree | 187 | 7.5 (5.1) | 64 (34.2) | 1.13 (0.88-1.47) | 1.12 (0.87-1.46) |
| 2-3rd degree | 359 | 8.6 (5.7) | 117 (32.6) | 1.02 (0.84-1.24) | 1.02 (0.84-1.25) |
| EDSS 4 | | | | | |
| Heredity | N | Years (SD) | Outcome (%) | HR (95% CI)^1^ | HR (95% CI)^2^ |
| No known family history | 2147 | 10.1 (5.8) | 335 (15.6) | 1.0 (reference) | 1.0 (reference) |
| 1st degree | 187 | 9.4 (5.5) | 25 (13.4) | 0.91 (0.61-1.37) | 0.88 (0.59-1.33) |
| 2-3rd degree | 359 | 10.3 (5.5) | 47 (13.1) | 0.85 (0.63-1.16) | 0.85 (0.62-1.15) |

CDW=confirmed disability worsening; EDSS=expanded disability status scale; HR=hazard ratio; CI=confidence interval; BMI=body mass index; DMT=disease-modifying therapy; ^1^adjusted for age and sex; ^2^adjusted for age, sex, ancestry, education, calendar year of diagnosis, disease duration, baseline EDSS, smoking, BMI, and DMT exposure.

eTable 3. Sensitivity analyses of disease outcomes with second- and third-degree family history analyzed separately

| Outcome | 1st degree relative | 2nd degree relative | 3rd degree relative |
| --- | --- | --- | --- |
| N | 222 | 146 | 303 |
| CDW | 1.10 (0.90-1.35) | 1.00 (0.79-1.26) | 1.05 (0.88-1.25) |
| EDSS 3 | 1.19 (0.92-1.56) | 1.04 (0.77-1.41) | 1.03 (0.80-1.32) |
| EDSS 4 | 0.96 (0.64-1.45) | 1.05 (0.68-1.61) | 0.74 (0.49-1.13) |
| New MRI lesions | 1.10 (0.90-1.33) | 0.87 (0.69-1.10) | 0.99 (0.84-1.18) |
| Cognitive decline | 1.05 (0.79-1.41) | 0.99 (0.70-1.40) | 0.90 (0.70-1.16) |
| MSIS-29 physical | 1.01 (0.80-1.26) | 1.07 (0.81-1.40) | 0.98 (0.81-1.20) |
| MSIS-29 psychological | 1.04 (0.85-1.28) | 0.77 (0.59-1.00) | 0.96 (0.81-1.15) |

CDW=confirmed disability worsening; EDSS=Expanded Disability Status Scale; MRI=magnetic resonance imaging; SDMT=Symbol Digit Modalities Test; MSIS-29=Multiple Sclerosis Impact Scale-29; HR=hazard ratio; CI=confidence interval; BMI=body mass index; DMT=disease-modifying therapy. Values are adjusted HRs (95% CI). Participants with no known family history of MS was the reference group. Models were adjusted for age, sex, ancestry, education, calendar year of diagnosis, disease duration, baseline EDSS, smoking, BMI, and initial DMT. The analysis of cognitive worsening was additionally adjusted for baseline SDMT score, and analyses of MSIS-29 worsening were additionally adjusted for the corresponding baseline MSIS-29 score.

eTable 4. Adjusted differences in longitudinal change at 5 years in SDMT and MSIS-29 scores according to family history of MS.

| Outcome | Family history of MS | N | Difference (95% CI) | P | P for time interaction |
| --- | --- | --- | --- | --- | --- |
| SDMT | No known family history | 1808 | Reference |  | 0.33 |
|  | 1^st^ degree relative | 183 | -0.62 (-1.89, 0.65) | 0.34 |  |
|  | 2/3^rd^ degree relative | 383 | 0.58 (-0.33, 1.50) | 0.21 |  |
| MSIS-29 physical | No known family history | 1885 | Reference |  | 0.74 |
|  | 1^st^ degree relative | 195 | 0.24 (-1.63, 2.10) | 0.80 |  |
|  | 2/3^rd^ degree relative | 394 | -0.06 (-1.41, 1.30) | 0.93 |  |
| MSIS-29 psychological | No known family history | 1885 | Reference |  | 0.94 |
|  | 1^st^ degree relative | 195 | -0.5 (-2.61, 1.61) | 0.64 |  |
|  | 2/3^rd^ degree relative | 393 | -0.20 (-1.73, 1.32) | 0.79 |  |

Values represent adjusted mean differences in change from baseline at five years, estimated using linear mixed-effects models with participant-specific random intercepts and slopes. No known family history was the reference group. Models were adjusted for age, sex, ancestry, education, calendar year of diagnosis, disease duration, baseline EDSS, smoking, BMI, and DMT exposure, and the baseline score of the respective outcome. Positive estimates indicate greater improvement for SDMT and greater worsening for MSIS-29. P for interaction tests whether longitudinal change differed according to family history group.
